# Connecting the Brain to Itself through an Emulation

**DOI:** 10.3389/fnins.2017.00373

**Published:** 2017-06-30

**Authors:** Mijail D. Serruya

**Affiliations:** Neurology, Thomas Jefferson UniversityPhiladelphia, PA, United States

**Keywords:** brain-computer interface (BCI), brain machine interface, organoid, whole brain emulation, medical devices, artificial intelligence, neuroprosthetics

## Abstract

Pilot clinical trials of human patients implanted with devices that can chronically record and stimulate ensembles of hundreds to thousands of individual neurons offer the possibility of expanding the substrate of cognition. Parallel trains of firing rate activity can be delivered in real-time to an array of intermediate external modules that in turn can trigger parallel trains of stimulation back into the brain. These modules may be built in software, VLSI firmware, or biological tissue as *in vitro* culture preparations or *in vivo* ectopic construct organoids. Arrays of modules can be constructed as early stage whole brain emulators, following canonical intra- and inter-regional circuits. By using machine learning algorithms and classic tasks known to activate quasi-orthogonal functional connectivity patterns, bedside testing can rapidly identify ensemble tuning properties and in turn cycle through a sequence of external module architectures to explore which can causatively alter perception and behavior. Whole brain emulation both (1) serves to augment human neural function, compensating for disease and injury as an auxiliary parallel system, and (2) has its independent operation bootstrapped by a human-in-the-loop to identify optimal micro- and macro-architectures, update synaptic weights, and entrain behaviors. In this manner, closed-loop brain-computer interface pilot clinical trials can advance strong artificial intelligence development and forge new therapies to restore independence in children and adults with neurological conditions.

## Introduction

Large-scale neural models primarily seek to identify the operating principles of the mammalian brain. “Whole-brain emulation” (WBE) can be seen as the pinnacle of such neural modeling efforts, comprising an attempt to recreate the number of neurons and synapses, of the human brain in a realistic neuroanatomical configuration.

One of the goals of the Human Brain Project is to develop the infrastructure capable of simulating a draft human brain model based on available experimental data, including the hardware and software to make it possible to simulate such a large-scale model, store and analyze its output, and control the simulation (Tiesinga et al., 2015). The human brain comprises an estimated 8.6 × 10^11^ neurons and approximately 10^14^ synapses presenting a formidable simulation challenge (Fornito et al., 2015).

In digital simulations, the dynamics of neuronal models are encoded and calculated on general purpose digital hardware, while in “neuromorphic” firmware, the dynamics of the neural systems are expressed directly on an analogous physical substrate. Models can be tailored to address brain dynamics from a wide range of spatial and temporal scales, from intracellular ion channel currents to million-neuron volume averages. While improvements in hardware have enabled increasingly sophisticated digital software and analog firmware models, emulating an entire human brain remains a significant challenge.

In addition to “whole-brain emulation,” bioengineering techniques continue to make progress toward “whole-brain recapitulation” in which tissue culture techniques applied to human pluripotent stem cells can be used to forge cerebral organoids (Lancaster and Knoblich, 2014; Chen et al., 2016). These organoids are three-dimensional constructs that can be coaxed into forming a structure mimicking the fetal telencephalon, or into particular neural tissue substructures (e.g., the midbrain Jo et al., 2016). Organoids, stacked cultures (organotypic slice, dissociated, micropatterned), and other three dimensional neural constructs could be used as “biological hardware” to achieve a kind of whole brain emulation (Pan et al., 2015; Albers and Offenhäusser, 2016).

In parallel with the development of large-scale neural models and organoids, research in neuroprosthetic devices have advanced our ability to record and stimulate ensembles of neurons in the mammalian brain for months and years at a time (Suner et al., 2005; Flesher et al., 2016). While the goal of neural modeling may be to better understand the operation of the brain and create strong artificial intelligence, neuroprosthetic devices are intended to treat human neurological disease and injury. In this review, an approach to developing whole-brain emulation and neuroprosthetic interventions together to enhance each other will be discussed. Whole-brain emulation, linked to a patient via a multi-site brain-computer interface device, could afford a versatile restorative potential significantly beyond what were possible with more narrowly specified decoder-effector systems currently being investigated in pilot human clinical trials.

Linking the real, human brain to one or more brain-inspired devices can be seen as a culmination of a neuroengineering trend to interface living neural tissue (*in vitro* or *in vivo*) to artificial entities (Vassanelli and Mahmud, 2016). These neurohybrid systems comprise at least one natural and at least one artificial entity and lay the ground work to create both “living robots” (such as small mobile robot reciprocally linked to an *in vitro* preparation of invertebrate motor ganglia) and “intelligent neuroprosthetics.” Even a brain linked to a living construct (or two real brains linked together) can be considered a neurohybrid because an artificial device is needed at some point to connect the two systems. Actuators or stimulators are needed to “write” information “into” living neural tissue, and sensors are needed to “read” signals “out” of the neural tissue.

For the purposes of clarity and consistency, a descriptive convention is proposed where {brain} refers to the patient's brain, {brain′} refers to the primary whole brain emulation (WBE), and where {x} refers to the actual region “x” in the person's brain, while {x′} refers to an emulated or external biologic counterpart intended to recapitulate that region's function.

## Minimal constraints for linking the brain to emulations

**Figure 1:**
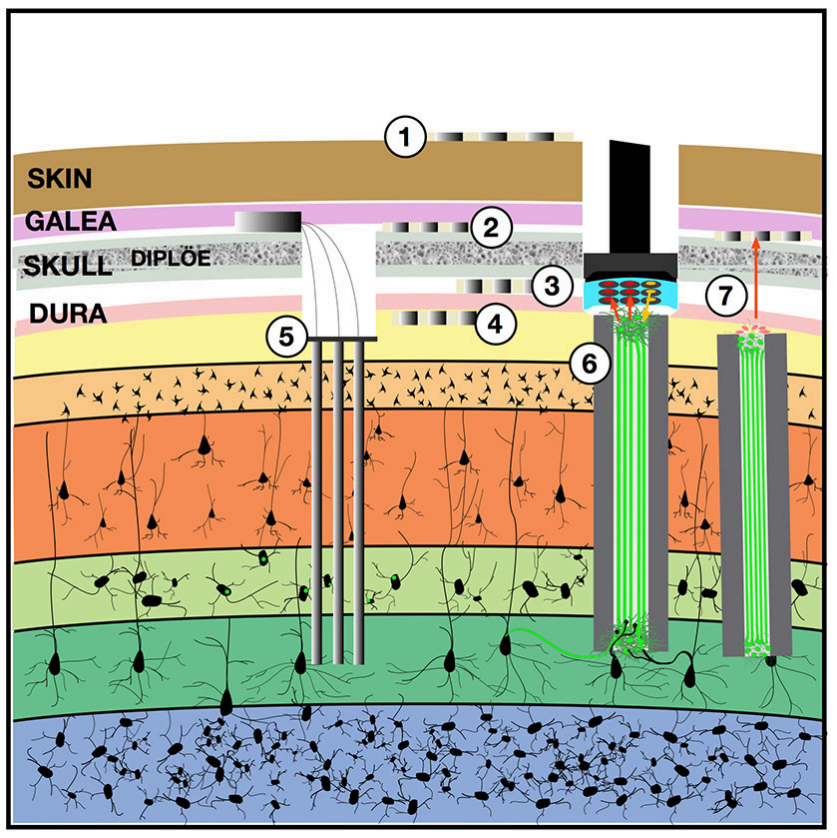
Implantable devices to chronically stimulate and record from the brain. Both low-impedance macro-electrodes and higher-impedance more densely packed electrodes can be used for electroencephalography (EEG) with contacts placed on the (1) scalp, (2) implanted in the subgaleal space without breaching the skull, (3) in the epidural space, or (4) in the subdural space where they may also be termed the electrocorticogram (ECoG) or micro-ECoG (Yu et al., 2016). These electrodes can capture local field potentials and can also be used to pass electrical current. To record ensembles of single units, (5) multi-electrode arrays can be implanted into the cortex, with or without integrated optical fibers. One solution to the apparent biological instability of rigid microelectrodes chronically implanted into the parenchyma, is to create a (6) “living electrode” comprising autologous neurons seeded within an agarose minicolumn that itself can be stereotactically implanted (Struzyna et al., 2015; Adewole et al., 2016). This three-dimensional living construct can both send axons to stimulate surrounding cortex and receive synapses to capture local activity and transmit this to an aggregate on the cortical surface where a planar optoelectronic array can reciprocally relay recordings and stimulation triggers with an external computer. The externalized aspect of the living electrode (7) could be capped with myocytes to achieve biopotential amplification such that signals could be captured by a subgaleal grid. That grid could in turn wirelessly broadcast signals to external computers. The subgaleal grid could also achieve stimulation of the brain, to provide input and feedback, by transmitting microcurrents to ephaptically modulate the living electrode cap in a manner analogous to the ampullae of Lorenzini in chondrichthyes, chondrostei and teleost fish and monotreme mammalian electroception structures.

Tens to hundreds of individual neurons can be recorded chronically from the mammalian brain, including humans, using implanted microelectrodes, with an apparently inevitable signal loss with time (Suner et al., 2005). Electrical stimulation can also be performed chronically, such as with deep brain stimulation and cochlear implants, and lacks the spatial specificity afforded by recording due to the biophysics of current spread, safety limitations imposed by charge density and device biostability, and the microanatomy of fibers of passage. Advances in microstimulation, optogenetics, endovascular recording, ultrasound neural dust, magnetothermal nanoparticles, genetically-encoded contrast, and the use of biological construct “living electrodes” to mediate the brain-device interface, may ultimately achieve lifetime recording and stimulation of ensembles of individual neurons (Watanabe et al., 2009; Shapiro et al., 2010; Wang et al., 2012; Seo et al., 2013; Chen et al., 2015; Adewole et al., in review). Existing stimulation/recording systems, and a hypothetical system that could be scaled up in which hundreds to tens of thousands of individual neurons could be bidirectionally addressed, via recording and stimulation, in a human brain across a lifetime, are shown in Figure 1.

A fundamental hypothesis of this review posits that for the human brain to be connected to one or more synthetic constructs in a clinically and behaviorally useful manner, the device(s) chronically implanted into the human brain must achieve the following requirements: (1) the action potentials of individual neurons must be recorded (extracellular), (2) the same individual neurons must be recorded continuously across the lifetime, (3) the device must be capable of stimulating ensembles of the same sets of individual neurons across the lifetime, (4) neurons from a variety of anatomical locations throughout the brain must be recorded and stimulated, (5) in addition to unit activity, the device must continuously record local field potential over a range of spatial scales. A set of interface parameters expected to be needed to link the human brain with an external emulation is summarized in Table 1. The lower limit on the number of neurons to be addressed, distributed throughout the brain, is estimated to be in the hundreds, with optimal brain-emulation function likely requiring thousands to tens of thousands. Given that there are ~180 anatomically and functionally distinct cortical “parcels” (Glasser et al., 2016), and given that approximately a third are likely to be inaccessible to interface implantation, due to surgical and vascular constraints, and given that a minimum of seven neurons suffice to decode behaviorally useful data from a putative parcel (Serruya et al., 2002), a minimum number of several hundred (840), can be estimated as a lower limit. That said, it is unlikely that chronic single-unit-fidelity recording/stimulation of millions of neurons were necessary. The minimal data acquisition and stimulation window is anticipated to be in the 50–100 ms range, such that spike counts in those windows would suffice (Oram et al., 2001; Stüttgen and Schwarz, 2008). The utility of the brain-emulation system would be expected to fall off dramatically with acquisition windows longer than 100 ms (Averbeck and Lee, 2003). While windows less than 50 ms might afford certain advantages, it is expected that these advantages would only be afforded for neurons recorded in certain areas (e.g., early sensory cortices) (Arabzadeh et al., 2006). Ideally there should be zero delay between the living brain and the emulation. The instant that action potentials have been counted in a 50–100 ms window from the recording at a particular channel within the real brain, this data should be available to the emulation.

**Table 1:** Hypothetical minimal constraints for chronically implanted neural sensor-actuators to link the brain to a whole brain emulation.

**Feature**	**Minimal**	**Optimal**
Number of single-units to record	500	500,000
Number of LFP channels	10	5,000
Time window	100 ms	50 ms
Anatomical sites	M1, PM, S1, A1, V1, BA10, BA46, DLPFC, TPJ, PPC, angular gyrus	Fusiform (vWF), pulvinar, thalam-retic nuc, striatum, GP
Data types	Spikes, LFPs	Spikes, LFPs, ECG, RR, GSR, kinematics, user inputs, audio, visual

Additional data streams that would augment the brain-emulation system, and yet would not be absolutely required, would include autonomic data (heart rate, heart rate variability, respiratory rate), sensory data (images recorded from cameras mounted on glasses, sounds recorded from microphones worn at the ears), kinematic data (accelerometers and gyroscopes worn on the body), user inputs (mouse and keyboard at a computer, tongue taps to tooth/palate sensors, sip'n'puff controllers, EMG sensors, and micromechanical switches).

The number and choice of anatomical sites in the human brain in which to implant bidirectional interfaces will rely on pre-operative characterization of a patient's specific lesions, deficits, surgical constraints, and rehabilitation goals. Hence for a person with a focal lesion (e.g., middle cerebral artery infarction), interfaces implanted around the lesion, and possibly in homologous cortical areas in the intact contralateral hemisphere, may be more logical choices than randomly selecting targets. For patients with conditions that affect the brain more diffusely (e.g., traumatic brain injury, multiple sclerosis, and neurodegenerative conditions), a larger number of anatomical targets may be needed. Rather than simply lay a uniform meshwork across the cortex and select spatially equidistant target sites, anatomical site selection should rely on the known function and connectivity of target areas. Functional MRI (including resting state-derived functional connectivity), diffusion tensor tractography, magnetic resonance elastography, transcranial magnetic stimulation, electroencephalography, neurological exam and formal neuropsychological testing could be integrated to define implant targets.

## Substrates for whole brain emulation

Given continuous, real-time bidirectional record/stimulate access to tens of thousands of individual neurons in a patient's brain, the clinician will require a principled strategy to identify a particular substrate for the linked emulated brain, and to optimize the potentially limitless parameters available to adjust in that brain emulation. Substrate options include digital simulations, neuromorphic firmware, biological constructs, other brains (e.g., of a service animal or another human being implanted with bidirectional interfaces), or a combination of all of these.

### Digital

Partial differential equations can be used to model cellular level biophysics, membrane potential, ionic concentrations, neuron geometry, and molecular cascades, at multiple time scales. The Blue Brain project started with modeling a single cortical column, using thousands of compartments per cell (Markram, 2006). Large scale thalamocortical models, with millions of multi-compartment spiking neurons, and billions of synapses, have been modeled in software, capturing receptor kinetics, short term plasticity, and long term spike timing dependent synaptic plasticity (Izhikevich and Edelman, 2008). Yet a one second simulation of 22 million neurons with 11 billion synapses in layer 2/3 took an IBM Blue gene supercomputer over 1 h, clearly demonstrating that such an approach would not be feasible for real-time linkage to a living, real brain (Djurfeldt et al., 2008). For the brain emulation to effectively be integrated with the patient's brain, it must operate on the same time scale. That said, digital models likely offer the greatest flexibility to explore model parameters and numerous open-source libraries are available to accelerate large-scale neural modeling that could lay the basis for WBE (Ames et al., 2012; Sanz Leon et al., 2013; Bekolay et al., 2014; Freeman et al., 2014; Vitay et al., 2015; Cheung et al., 2016; Ulloa and Horwitz, 2016).

More recent work has made progress at real-time simulation of 50,000 neurons, with 50 million synapses, at a time (Sharp et al., 2014). Chains of graphic accelerator cards could enable the digital simulation of millions of Hodgkin-Huxley spiking neurons simultaneously (Yavuz et al., 2016). Rather than modeling neurons specifically, multi-input/multi-output (MIMO) nonlinear dynamic models have been used to capture underlying spike train-to-spike train transformations between areas within the hippocampus and neocortex (Berger et al., 2011; Hampson et al., 2012). These MIMO models have been applied to ensemble recordings to extract patterns of firing related to successful task performance and have been used in real-time to facilitate and recover performance when administered to the same locations as patterns of electrical pulses delivered back into the brain (Deadwyler et al., 2017).

Numerous reasons why higher brain functions would not be expected to spontaneously emerge from digitally-based simulations (syntactic Turing machines) are described elsewhere (Cicurel and Nicolelis, 2015). The key utility in digital attempts at whole brain emulations would lie not in their fidelity of mimicry of a biological brain, and rather in their flexibility in exploring connectivity patterns when linked reciprocally to a living, human brain with an explicit goal of restoring day-to-day function.

### Analog

The most studied neuromorphic hardware approach to modeling neural systems is based on very large scale integration (VLSI) technology (Indiveri et al., 2011). In addition, memristors (Strukov et al., 2008), carbon nanotubes (Joshi et al., 2011), and organic nanowires (Xu et al., 2016) have been explored for their synaptic-like plasticity. Unlike logic gates in a digital computer, VLSI neuromorphic chips rely on silicon neurons: analog electronic circuits of transistors that mimic the ion-channel properties of a real neuron with configurable interconnectivity. Once the balance between cell count and connections per cell can be adjusted to simulate multiple cortical areas in real time, the arrangement can scale up to include tens of millions of neurons (Merolla et al., 2007; Silver et al., 2007). Analog substrates could also recapitulate their own equivalent of the ephaptic and capacitive coupling interactions linking local field potentials and membrane potential biases altering spike timing that may be crucial for a priori active model representation of the world and dynamic, integrative exchange with the environment (Cicurel and Nicolelis, 2015).

### Biologics

The connectivity principles of the real-synthetic interface could generalize across a wide range of synthetic instantiations. In addition to digital simulation large-scale neural models and neuromorphic analog circuits, the “emulated brain” module could also comprise biological substrate, such as dissociated cultures or organotypic slices grown chronically on multi-electrode arrays in perfusion chambers (Killian et al., 2016), or neural organoids (themselves implanted with living electrodes) sustained either *in vitro* incubators, or implanted into the patient's body. Cerebral organoids appear to recapitulate the endogenous developmental program, and can give rise to developing cerebral cortex, ventral telencephalon, choroid plexus and retinal identities, among others, within 1–2 months (Lancaster and Knoblich, 2014; Chen et al., 2016).

In terms of clinical application, *in vitro* culture systems are fragile and, should a patient become dependent on the neural function they restore, could pose a risk if they themselves became compromised by infection or mechanical breakdown. For development purposes, *in vitro* culture systems may be more versatile, allowing investigators to systematically alter tissue variables and explore how it altered behavior, while for long-term clinical applications might rely on self-contained organoids (that ultimately would have their own blood supply) that could be implanted into the patient.

In certain cases, temporary use of an *in vitro* biological intermediary can be used to identify what transfer function it performs transforming inputs into outputs, and recast that function as mathematical equations that can be performed at the same speed in the substrate of hardware. This has been done with lamprey eel motor ganglia sustained in *in vitro* and linked reciprocally to a small mobile robot; upon deriving the ordinary differential equations that represented how the ganglia transformed inputs into outputs, the actual living ganglia could be discarded and replaced with hardware performing the equation's operations (Reger et al., 2000). The drawback to this approach is that the transfer function may be over fit to the conditions provided during the training and may thus not generalize the intrinsic abilities that the original biological system could provide.

## Connecting the brain to itself through an emulation

If we posit the technology available to record and induce trains of action potentials in tens of thousands of individual neurons at numerous locations in the human brain, then we can consider linking these neurons to a variety of synthetic architectures. While a synthetic system could continue to get its own inputs (e.g., from a camera) and generate its own outputs (e.g., to a robotic arm), its neural components could receive ongoing input from neural counterparts living within a patient's brain. Spike train data emerging from the brain could be used as inputs to modeled or cultured neurons; likewise the activity of modeled or cultured neurons could be used to trigger and specify the stimulation of neurons living within the brain.

**Figure 2:**
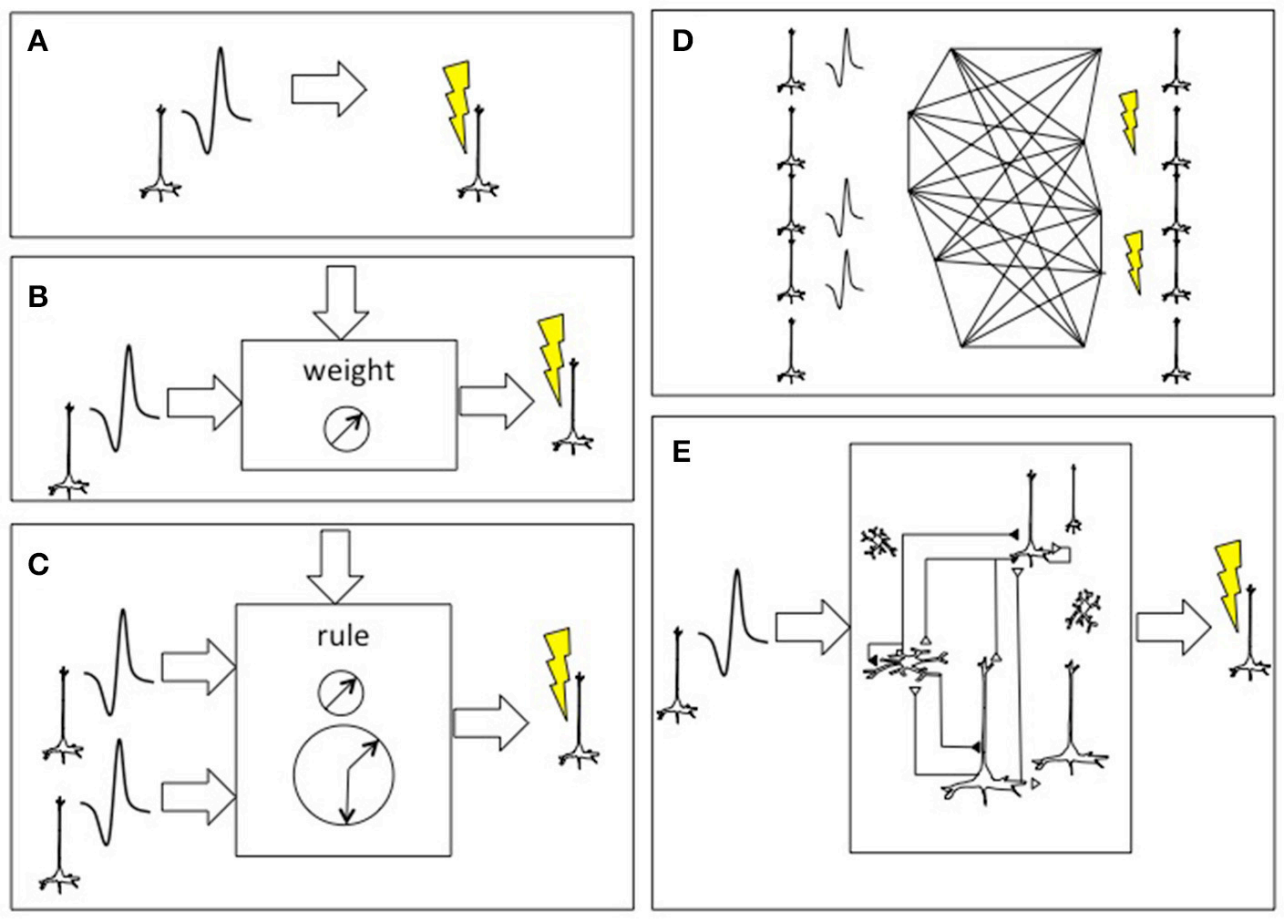
Approaches to connecting the brain to itself. **(A)** In spike-triggered microsimulation, the detection of an action potential generated by a neuron recorded by an implanted microelectrode triggers focal microstimulation at microelectrodes located in another area of cortex (Jackson et al., 2006). **(B)** Rather than triggering stimulation in a direct, linear manner, the relationship between the spike detection and microstimulation could be governed by a weighting rule meant to emulate features of synaptic plasticity. **(C)** The external system could be altered to incorporate spike trains from two or more neurons and use spike-time dependent synaptic plasticity learning rules, including temporal coincidence detection, in order to trigger target. **(D)** As the BCI were able to record and stimulate more individual neurons, the external system could deploy learning rules to update the synaptic weight between all potential pairs, shown here on an image based on William James' hypothesis about neural process interaction (James, 1890). **(E)** Beyond direct mapping of recording to stimulation, or an interposed simplified neural network, more realistic cortical simulations could be used with the premise that the emulated cortex would be capable of performing its own “canonical” operations and would hence add a computation beyond connectivity (Nelson, 2002; Kouh and Poggio, 2008; Miller, 2016). Figure reprinted from Serruya and Kahana (2008) with permission from Elsevier.

In the simplest instantiation of a living-synthetic reciprocal neural interface, the presence of an action potential recorded from a microelectrode in the living brain triggers microstimulation of a focal neural population at another location within that same living brain. The external system artificially instates a physical connectivity between two neural populations. The linkage between spike trains streaming out of the brain and the triggers for stimulation of distinct neurons within the brain can become increasingly elaborate, as shown in Figure 2 (Serruya and Kahana, 2008). As the number of independent neurons grows, the system evolves from a learning rule or glorified voltage clamp into substrate expansion, where the intervening processing- performed on simulated, synthetic or ectopic biological neurons- ought to achieve novel computation not otherwise possible in the endogenous brain itself. The synthetic intermediary can be a large-scale neural model, and ultimately a whole-brain emulation that both parallels and reciprocally interacts with a patient's brain in real-time.

At its most basic level, the parallel whole brain emulation could restore neural function following injury and disease by providing an auxiliary pathway linking areas that were disconnected by the disease process. This homologous brain (whether emulated as digital software, neuromorphic firmware, or bioengineered neural constructs) would function continuously in parallel with the patient's brain and would compensate for disconnections within the patient's brain through connections within itself. Given a disruption between primary motor {M1} and sensory cortices {S1} (such as due to a stroke, mass lesion or demyelination), interfaces implanted in those two cortical areas could use “blind” mapping rules, e.g., spike triggered stimulation, so if a neuron in M1 fires, it stimulates a neuron in S1, thus instantiating a virtual U-fiber fascicle linking the two. The alternative is to interface {M1} and {S1} separately to the auxiliary homolog and such that {M1′} and {S1′} have intact connectivity. While one could create non-human animal model to compare these two options (direct {M1 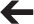
′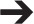 S1} vs. WBE-mediated indirect {M1↔M1′↔S1′↔S1}), the model system would be very contrived and over fit and it would be challenging to generalize given the heterogeneity of individual human brains and the heterogeneity of disease.

The availability of spike train data streaming from multiple neurons recorded simultaneously at multiple sites within the brain, and the ability to selectively stimulate particular sets of neurons, immediately presents a design question of how to assign, or map, this activity reciprocally onto the synthetic construct. For neuromotor prosthetic decoding, all neural signals are analyzed by a dedicated decoder. While this approach is appropriate for achieving a particular functional goal (e.g., motor intent to drive a spinomuscular stimulator, or decoding speech for communication), for a large-scale neuroprosthetic with an order of magnitude or more of ensemble data, different subsets of this data could be mapped onto different targets in the synthetic construct.

## Mapping the brain and the emulation to each other

In a straightforward ***homologous “afferent copy” mapping***, real neural activity recorded from a given brain area (e.g., primary motor cortex layer V) would be used to “stimulate” the synthetic neurons in the matching brain area of the model (e.g., the synthetic excitatory neurons of layer V of motor cortex in the model). In a ***directive transfugal mapping***, the activity from the real brain would be used to drive activity of its homologous target (e.g., real primary motor cortex layer V neurons driving synthetic neurons of simulated spinal ventral horn, red nucleus, basal ganglia and cerebellum). In a ***convergent integrative mapping***, activity from numerous neurons recorded at numerous interface sites throughout the patient's brain, may be used to stimulate higher-order cortical areas in the model, to promote the emergence of abstract categories via convergence of information across modalities (Mesulam, 1998). These higher-order areas are known to serve an integrative function, including prefrontal cortex, angular gyrus, and entorhinal cortex. This integrative approach could also be performed in the other direction, with “recordings” from a wide variety of areas in the emulation being assigned to higher-order cortical areas in the patient's brain. In a ***divergent duplicative mapping***, recordings from a given neuron or a single cluster of neurons in the real brain are broadcast in replicate to numerous targets in the synthetic brain model. The same mapping rules can apply in reverse, with “recordings” of synthetic neurons in the simulated brain being used to target the timing and location of optical or electrical stimulation of real neurons across widespread sites in the real brain.

Granting that a single, static “normal” structural-functional brain state does not exist, there may be merit in comparing a patient's unique endogenous functional connectivity to an average derived from a normative database of age-matched healthy controls. The purpose of identifying the discrepancies between the patient's pattern and this normative average is not to foist a preconceived notion of normality, and instead is to guide a process that recapitulates gross structural linkages (e.g., the two hemispheres linked through the corpus callosum) and presumed “healthier” graph theoretic properties (e.g., coaxing the inter-regional connectivity pattern of the patient's brain to adopt clustering and global efficiency properties seen more frequently in healthy individuals of the same age). This ***connectivity normalization mapping*** may be achieved either by {brain}-{brain} connections via the BCI (“brain-brain interface” or “brain-computer-brain-interface,” BCBI), or via using the WBE in its entirety as an auxiliary parallel homolog to the real brain. The normalization must address the von Monakow diaschisis of a given lesion (i.e., the disruption of intact regions via linkage to lesions that cause net deafferentation of excitatory input to the remote intact area) (Alstott et al., 2009). Pre-surgical mapping of anatomical connectivity and activation patterns can inform both the anatomical target of sensor-stimulator implants and the calibration approach to cycle through tasks whose performance was to be optimized.

While the crucial mapping between the real and synthetic brains is achieved at the level of ensembles of spiking neurons, additional local field potential, autonomic, endocrine, kinematic and other data types can be deployed reciprocally. Local field potentials (LFP) recorded at subdural, epidural or subgaleal arrays can capture activity from a wider spatial-anatomical range than the device-constructs used for unit recording-stimulation (see Figure 1). Two obvious applications of LFP data include: (1) spectrotopic mapping, and (2) arousal state synchronization.

**Figure 3:**
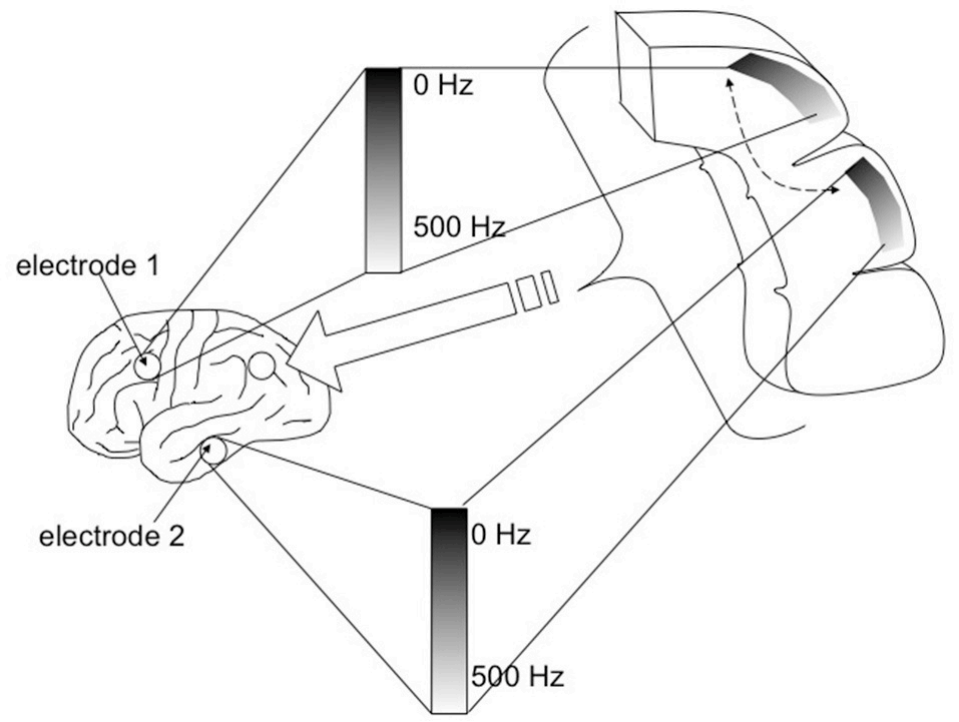
Spectrotopy: an approach to mapping the brain's own power spectral data on to itself. Just as primary auditory cortex deploys a tonotopic mapping where different sound frequencies are arranged topographically along the cortical surface, so too power spectral features of neural activity itself could be mapped along emulated cortex that in turn would send inputs back into the real brain. While the brain localizes sound in three-dimensional physical space, capturing local field potentials from multiple brain areas entails higher dimensionality. Fortunately emulated cortex is free from the spatial anatomical constraints of real cortex, and could extend in different dimensions such that location along the emulated cortex would move along gradients indexing both frequency and real brain cortical origin. Each emulated minicolumn could represent both frequency and temporal phase features of the recorded activity. Figure reprinted from Serruya and Kahana (2008) with permission from Elsevier.

Spectrotopic mapping comprises the mapping of LFP power along a frequency axis realized along the simulated spatial domain of the neural model in a matter akin to auditory tonotopic mapping where LFPs are used instead of sound (Figure 3). In addition the frequency dimension (that may be mapped to a single spatial or simulated spatial dimension in the construct, much like tonotopy along the superior temporal gyrus), another dimension would reflect the anatomical origin of the LFP data, and could follow numerous mapping regimes including geodesic cerebrotopy or distance along a connectivity gradient (e.g., unimodal-to-heteromodal, or primary/concrete-to-default-mode/abstract).

Arousal state synchronization refers to capturing the focal and global state of the patient's brain (awake, drowsy, slow-wave sleep stages, rapid eye movement sleep, rotating waves, sleep spindles) and conveying this information to the large scale neural model so that it also “sleeps and dreams” along with the real brain (Muller et al., 2016). At a more granular layer, focal LFP recordings can also capture up/down states of cortex, and based on living electrode phenotype, extracellular concentrations of glutamate, acetylcholine and other transmitters to define cortical and corticothalamic states. In addition to recording neural activity from the cortex, bidirectional interfaces could be implanted into subcortical basal ganglia and thalamic nuclei to provide additional signals that reflect the brain's state of arousal and global processing (e.g., recordings from the nucleus accumbens and ventral pallidum could index the survival salience of a particular sensory impression).

One approach to linking the real brain to one or more synthetic emulations is to leverage brain areas known to be involved in synchronization of behavior between people, such as right temporoparietal junction and dorsolateral prefrontal cortex (Sänger et al., 2012; Tang et al., 2015). Likewise, spectrotopic mapping in the emulation, could have output hubs positioned at emulated cortices representing temporal and lateral parietal regions in the 6–12 Hz oscillatory range, both in terms of covarying amplitude (Dumas et al., 2010) and precise temporal phase synchronization (Lindenberger et al., 2009; Sänger et al., 2013).

The WBE has the potential to gain functional attributes beyond what were present in the patient's brain by increasing literal physical and emulated simulated distance (maps of maps), extrapolating from existing principles of distributed association networks in the human brain (Buckner and Krienen, 2013). If global connectivity of prefrontal cortex predicts cognitive control and intelligence (Cole et al., 2012), can one use BCI to increase global connectivity and consequently enhance cognitive control and intelligence?

Optimal nodes to link the real brain to the emulation include heteromodal multisensory areas (Bizley et al., 2016) and default mode hubs (van den Heuvel and Sporns, 2013; Margulies et al., 2016). The default mode network (DMN) comprises a group of brain regions (e.g., frontal gyri, retrosplenial cingulate, etc.) that deactivate during externally oriented tasks, and activate in introspective tasks, and that act as hubs integrating representational information across cortex. DMN hubs in the patient's {brain} hence serve as ideal targets to receive information from primary and secondary sensory and motor areas in the emulated {brain′}; likewise, the {brain′} DMN hubs can receive recordings from ensembles of neurons in the primary and secondary sensorimotor areas in the patient's {brain}.

An outstanding question is how the brain's own principles of connectivity and computation (Steyn-Ross et al., 2009; Moon et al., 2015; Turkheimer et al., 2015) can be distilled and then applied for (1) the design of the {brain′} itself and (2) the optimal linkage between {brain} and {brain′}. The optimal linkage includes both the input/output mapping between {brain} and {brain′} and also the temporal lags: the real human {brain} is already optimized to use geodesic distance for coincidence detection and various scales of temporal integration (Chapman et al., 2002; Rodgers et al., 2006; Seidl, 2014; Bastos et al., 2015; Zhang and Jacobs, 2015). Hence there may be cases where a temporal lag should be purposefully introduced in the {brain}-{brain′} linkage (in either direction). In addition to time lags, there may be other techniques of pre-processing data streams as they move back and forth between {brain} and {brain′}, such as being assembled explicitly into synfire chains (Wang et al., 2016).

## Emulation architecture

Given the selection of a particular substrate, the optimal architecture *within* the emulation must be identified. “Architecture” includes number of neurons, type of neuron model, number of compartments per model, number of synapses, synapse learning rule, circuitry within an area, connectivity between areas, inclusion of non-neural elements, and nonlinear dynamical components (Soriano et al., 2015).

Any given emulation architecture must balance the granularity of its modeling scale with processing speed. Even though a single pyramidal neuron, when modeled with thousands of synaptic inputs, can by itself exhibit sequence memory (Hawkins and Ahmad, 2015), the time to simulate multiple neurons at that level of detail would render the emulation too slow to meaningfully interface with the real brain. Simpler single-neuron models are more amenable to being scaled up and the resulting emulations manifest useful emergent properties: a 2.5 million spiking neuron model with three hierarchies modeled on simplified visual, motor, basal ganglia, and thalamocortical circuits, was able to select actions when given visual sequences as input and a modeled arm as output (Stewart et al., 2012). An alternative to simplifying the software-rendered model of individual neurons is to render the emulation in firmware: a neuromorphic model with sparse connections between simulated neurons, was able to perform real-time context-dependent classification of motion patterns observed by a silicon retina (Neftci et al., 2013).

Imaging, anatomical and modeling studies suggest the brain has a modular architecture at the scale of cubic centimeter regions and WBEs, with nearly two hundred distinct cortical “parcels” in each hemisphere (Brodmann, 1909; Economo and Koskinas, 1925; Bertolero et al., 2015; Glasser et al., 2016; Wang et al., 2016). To design an emulation architecture, the modeler must decide how many of these regions to emulate, in what detail to model each region, and how to connect them all to each other. There is likely some minimal level of detail to emulate a given region to recapitulate its unique processing abilities. Simply naming nodes in the emulation model “middle frontal gyrus” and “inferior frontal junction” will not magically endow the modeled region the structural and functional features of their intended eponymous region. The general design of all emulated “parcels” could be based upon a basic wiring diagram fundamentally unaltered across mammals and all cortical regions (Nelson, 2002). This putative “canonical” cortical microcircuit contains 400,000 neurons across six layers with a particular connectivity pattern between the excitatory pyramidal and inhibitory interneurons. This canonical circuit gives rise to computational operations including feedforward selectivity, divided normalization, recurrent gain, gain control, signed-like response, gaussian convolution, pattern recognition, and hierarchical temporal memory (Kouh and Poggio, 2008; George and Hawkins, 2009; Miller, 2016). The unique function of each cortical “parcel” likely arises from details of how its circuitry varies slightly from the canonical circuit, and the parcel's pattern of connectivity to subcortical structures other cortical regions.

The normal function of neocortex relies on its connections to subcortical structures including the basal ganglia, thalamic nuclei, amygdalae, hippocampal formations, the cerebellum and brainstem nuclei. To truly emulate the “whole” brain, a WBE should include these subcortical areas, and emulate the connectivity patterns they exhibit between each other and with cortex. Just as there appears to be a canonical circuit within neocortex, so too there appears to be stereotyped connectivity patterns within and between these subcortical structures, including cortico-basal-ganglionic (Lanciego et al., 2012) and thalamocortical loops (Llinás and Ribary, 1993).

The connectivity pattern directly between cortical regions can be based on large repositories of openly shared connectivity maps (Laird et al., 2011; Van Essen et al., 2013)^1^For the WBE to recapitulate and improve higher-order cognitive abilities, patterns of connectivity between emulated and real brain regions are expected to be crucial. The ability of an emulated lateral prefrontal cortex to recreate a gradient ranging from future-abstract goals to concrete-present-context needs (Nee and D'Esposito, 2016), will rely on the pattern by which it were connected to other emulated cortical regions and to numerous sites in the participant's real brain.

## Calibration and convergence

In the scenario that a human brain were reciprocally linked to a synthetic model, with thousands to hundreds of thousands of firing rate data streams flowing continuously between them, ensuring that the synthetic system converged on a behaviorally useful activity state would mark the next challenge. While spontaneous endogenous activity may suffice in certain cases, it is reasonable to hypothesize that calibration would be needed to march the brain through numerous tasks and space states in order to pull the synthetic system along with it so that the synthetic system could update its synaptic weights and connectivity patterns accordingly. This calibration phase may involve canonical tasks known to activate well-known discrete human brain activity patterns/behaviors, and may also be tailored to the particular deficits a given patient sought to overcome. By linking the WBE to an awake, behaving human being, the systemic identification of the emulation subcomponents (Koene and Deca, 2013) can include behavioral and psychophysics measurements.

Using canonical “sentinel tasks” investigators can also explore which synthetic brain model architectures (in terms of substrate digital/analog/biological, synaptic learning rules, circuit pathways, etc.) were most helpful. A “sentinel task” is defined as one in which an objective change in the person's behavior, or a consistent reported subjective change, were causally linked to the connectivity and intact functionality of the synthetic neural model system. For example, the delayed match to sample task has been deployed in rodent and non-human primates to assess the utility of implanted microelectrodes arrays reciprocally linked to a digital massive-input massive-output program (Hampson et al., 2012). A large suite of validated neuropsychological tests, each with available normative performance data, is available to probe different aspects of cognition (e.g., color-word interference, verbal learning tasks, line orientation). The clinician-investigator could compare the patient's performance at baseline and then with various parameters set in the linked WBE (e.g., to see if performance would rise from <0.1 percentile to 50th percentile or higher).

In addition to resting state (“non-task”), active tasks should be selected to ensure all of the known, consistent networks were activated (sensorimotor, visual, auditory, dorsal attention, ventral attention, alertness, salience, executive control, reward emotion, and language) (Damoiseaux et al., 2006; Cataldi et al., 2013). Orthogonal task design can be used to help how data variance can be explained by one variable vs. another (Courtney, 2012). Double dissociation and working at threshold (i.e., asymptotic performance of full engagement) can be used to deal with confounds of attention, motivation and difficulty. Block designs, m-sequences, and temporally independent components of tasks can be contrasted with one another (Ginsberg et al., 1987; Fetsch, 2016). Genetic algorithms can be used to generate task designs that are optimal for linear estimation in the presence of uncertainty in the noise autocorrelation structure (Liu, 2012), and naturalistic videos and tasks can evoke endogenous cortical ensemble sequences (Jones et al., 2007).

The ability to simultaneously record the activity of thousands to tens of thousands of individual neurons in a living human brain presents an enormous functional identification challenge. This investigator posits that the identification procedure must consider each neuron both as an individual and as node in an ensemble and that either approach alone risks missing crucial information. A trade-off exists between precision and the time required to map a receptive field for a given neuron. Manual, qualitative methods are fast yet impose a variable degree of imprecision, while quantitative methods are more precise and require more time. A rapid quantitative method for mapping visual receptive fields, named back-projection, could be adapted for other sensory modalities (Fiorani et al., 2014). A firing rate map, also termed a tuning curve, describes the nonlinear relationship between a neuron's spike rate and a low-dimensional stimulus (e.g., orientation, head direction, contrast, color). The closed-loop nature of BCI investigation, affords an opportunity to use Bayesian active learning methods, including a utility function that selects stimuli to minimize the average posterior variance of the firing rate map and analyze the relationship between prior parameterization, stimulus selection, and active learning performance (Park et al., 2014). Other approaches include the “model deterioration excluding stimulus test,” that identifies the contribution of stimulus to spiking activity while taking into account task-irrelevant intrinsic dynamics that affect firing rates (Kahn et al., 2015), stochastic gradient descent and generalized linear classification schemes (Meyer et al., 2015), and the use of non-Gaussian stimuli that facilitates the discrimination of spike-eliciting from non-spike-eliciting stimuli (Meyer et al., 2014).

Investigators can purposefully leverage the patient's brain's ability to learn novel tasks to dramatically constrain the convergence process from a combinatorial morass to a tractable calibration session. For neuromotor decoding, patients could be taught “neural gestures,” or “neural sign language” imagined or attempted discrete movements that could be assigned to effectors and could be multiplexed (e.g., an imagined lifting of a supine arm could be mapped to raising the volume, moving a scrollbar or cursor up, or increasing the amplitude of any abstract process). This may be construed as an analog to the stenographic “graffiti” used for the precursors of contemporary mobile smart phones (Butter and Pogue, 2002). Patterned stimulation contingent on neural activity and behavior can leverage innate plasticity to purposefully steer function to the anatomical sites where implanted recording-stimulating devices link to the WBE (Jackson et al., 2006; Rivera-Rivera et al., 2017). The full benefit of this reciprocal brain-WBE linkage may only be recognized in the setting of real-time evidence-based rehabilitation approaches, including adaptive cognitive remediation computer games, biobehavioral environmental home-based interventions with both the patient and caregivers, and iterative direct skill and cognitive training with a therapist (Gitlin et al., 2010; Tacchino et al., 2015; Winter et al., 2016; Charvet et al., 2017; Skidmore et al., 2017; Train the Brain Consortium, 2017).

## Aiming the attentional spotlight across more than one brain

Attention can be defined as the ability to select information relevant to current task and filter out the rest (Buschman and Miller, 2010). Just as attention serves to organize behavior and coordinate activity within a single human brain, so too attention systems should coordinate activity across the one or more emulations that the real brain were reciprocally connected to, and between the emulations and the real brain.

Optimal brain-emulation hybrid function may rely on literally linking the WBE to key attentional nodes in the real brain and on explicitly emulating the connectivity of hubs known to be crucial for attention. Potential approaches include:

- The WBE could be modulated by the bottom-up content-encoding driving system of the ascending reticular activating system, and the top-down contextual salience modulation from higher order prefrontal, parietal and limbic cortices (Mesulam, 2010; Kanai et al., 2015).
- Signatures of attentional modulation in the real brain could be used to increase the connectivity to nodes within the WBE that are active and relevant to the task, and decrease connectivity to nodes that are not (Alnæs et al., 2015).
- The tonic alertness of wakeful arousal could be “spread” from activity recorded from a right hemispheric fronto-parietal thalamobulbar network the real brain to diffuse one-to-many stimulation in the WBE (Sturm and Willmes, 2001).
- The WBE could have its own “simulated attention” function semi-autonomously such that cues derived from external sensors could induce a type of phasic alertness stimulating a left hemispheric frontoparietal network both in the WBE and the real brain (Sturm and Willmes, 2001).
- The WBE could derive attentional and arousal signals in the real brain from lateral prefrontal cortex to index midbrain dopamine input (Bahlmann et al., 2015), anterior cingulate to reflect salience (“search value,” “choice value”) (Kolling et al., 2016), and the pulvinar nucleus to flexibly link sensory stimuli to context-specific motor responses (Arend et al., 2008; Leh et al., 2008; Padmala et al., 2010; Wilke et al., 2010; Zhou et al., 2016) and counter inter-representational interference (Arcaro et al., 2015).
- The WBE could use signatures of alpha-band oscillations in the real brain sensory cortex, to toggle between multiple low-level perceptual representations streaming from external devices, and unified, broad attention (McMains and Somers, 2004).

The brain selects appropriate sensory inputs and suppresses distractors through a top-down prefrontal cortical (PFC) modulation of the thalamic reticular nucleus (TRN). One conception of attention derives from Crick's observation that, “if the thalamus is the gateway to the cortex, the reticular complex might be described as the guardian of the gateway” (McAlonan et al., 2006, 2008). This idea led to the metaphor of a “spotlight” of attention as a mechanism for behaviors such as the targeting reaction in which the eyes and head are moved so that an external target falls on the fovea (Sokolov et al., 2002; Frey et al., 2014). This behavior relies on coordinated activity of neurons in the premotor nuclei, superior colliculi and the reticular nucleus of the thalamus, and is accompanied by unique local field spectral signatures including correlated gamma activity in prefrontal and TRN (Buschman and Miller, 2010). With an interface implanted into the PFC, the range of this “spotlight” could be extended to the WBE's modeled TRN, either directly ({PFC} 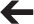 {TRN′}) or indirectly ({PFC} 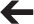 {PFC′} 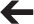 {TRN′}) (Zikopoulos and Barbas, 2006; Wimmer et al., 2015; Phillips et al., 2016).

## Strong artificial intelligence

Intelligence comprises the ability to acquire and apply knowledge. Artificial intelligence signifies human-built artifacts that exhibit this ability. In “strong” artificial intelligence, the system transitions from merely “simulating” a mind to actually “having” a mind, in the same sense human beings have minds (Searle, 1999). This distinction appears to rely on whether consciousness emerges, and may be irrelevant if the only metric were that a particular AI program functioned as intended (Russell and Peter, 2003). A WBE in a {brain↔brain′} poses a unique situation where the consciousness of the person ({brain}) could in principle “expand” to include the synthetic WBE ({brain′}) and where this person would be able to provide a report of the subjective experience of being linked to the emulation. While deep brain stimulators, cochlear implants and neuromotor prosthetics are such purpose-driven tools that the idea that their actual inert hardware would have a “mind” can be disregarded, WBEs could reach a level of complexity that they enter a qualitatively distinct regime.

The rationale why the coupled system could promote strong AI asserts that by having the synthetic model continuously “seeded” by actual neural data from a human brain that were already “embodied”(Yamada et al., 2016)—especially if the patient were engaged in a variety of quasi-orthogonal canonical tasks- will coax the model into state spaces that could never be achieved if the model resided in isolation and even if the model were trained using a contrived and quite circumscribed input/output arrangement (e.g., a single planar array of pixels for “visual” input and a simplified multi-jointed limb model for motor output). The reciprocal connection to the brain of an awake, behaving human being is expected to steer the parameters of the brain emulation into a more stable regime.

The “seeding” hypothesis posits that the WBE, once fully trained and updated via its ongoing reciprocal connectivity to a living, real brain, would be able to function independently (via its own external sensors and effectors) even after being disconnected to that living brain. The WBE thus would transform from only being an “auxiliary brain” medical device for a patient, and would become a potentially autonomous agent when evaluated in isolation, independent of the patient. The potential consciousness of the WBE would not be necessary for the WBE to be clinically useful to a person whose brain were reciprocally linked to treat a neurological condition, and instead might be an unintended emergent property. The consciousness of the WBE, upon disconnection from a real, human being, could serve useful simulation functions to serve the person's rehabilitation upon reconnection, and would likewise raise questions of autonomy and other ethical quandaries that are beyond the scope of this paper.

## Beyond a single emulation

The ability to link the human brain to an external construct, opens the possibility of linking the brain to more than one WBE and to more than one type of architecture. To determine the optimal approach to linking the real human brain to one or more brain emulations, a larger question of what direct brain-computer interfaces could afford must be addressed. This question at its heart asks how to merge the best of computers and to the best of the brain. To restore and augment human function, is it better to link it to a brain-like synthetic emulation, or is it better to link it to the computer more directly?

One direct linkage can be conceived as a “brains-up display,” playing on the analogy of “heads-up displays” where images are displayed close to the eyes. In principle, a BCI could be used to induce perception of sound, images and tactile sensation by directly stimulating auditory, visual and somatosensory areas to replace audio speakers, display screens and haptic gloves, and could record from motor and language areas to derive motor commands and enter text to replace mouse, keyboard and voice recognition. Beyond this “surface brains-up” using cortices “one-synapse-in” from the periphery, a “deep brains-up” system could in principle afford a more intuitive experience by interfacing with higher-order areas. Hence instead of entering input text through a motor cortical derived cursor/keyboard, one could decode words and their meanings from ensembles in left inferior frontal and posterior superior temporal gyri (Wang et al., 2011).

**Figure 4:**
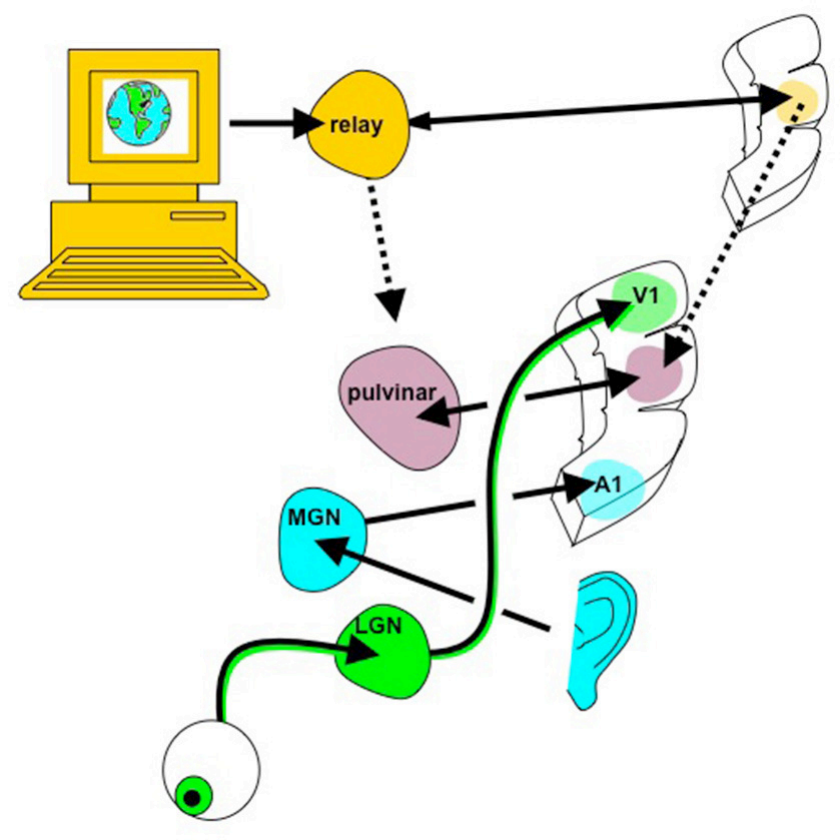
Whole brain emulation to mediate digital information exchange. Instead of using the existing sensorimotor apparatus for internet browsing (eyes to see the screen, hands to control mouse/keyboard), in principle digital information, including abstract data structures that have no obvious sensory correlate, could be navigated via a customized “internet organ” (akin to the retina or organ of Corti) leveraging the numerous pattern recognition computational abilities of neocortex. The emulated {thalamocortical'} circuit processing this digital data could be reciprocally linked with real thalamic and cortical areas known to already process multi-modality sensory information, such as the pulvinar, angular gyrus, and tempoparietal junction. Figure reprinted from Serruya and Kahana (2008) with permission from Elsevier.

While this approach could certainly afford a more immersive, dynamic user experience, it does not axiomatically provide any computational benefit. It is unclear if the ability to silently use web browsing and texting features, and to receive vivid audiovisual reminders, would compensate for the fundamental memory, executive function and other deficits that afflict certain people with neurological injury. An outstanding question for the WBE is whether it could provide a better intermediary between the ensemble spiking “neural-ese” of the brain and the digital language of computers. Could the canonical neocortical circuit be altered to create new cortical “parcels” to optimize data exchange with artificial processors (e.g., neural-to-binary or neural-to-qubit)? This highlights the need to identify the relative strengths and weaknesses of brains and computers so to identify what should be built into the WBE. Does it make sense to make the biological brain more computer-like and the computer-based system more brain-like, and is there a happy medium that can be bridged by a WBE that has features of both (Figure 4)? Computers are better at sequential logic operations and deep search in unstructured data, while biological brains are better learning structure from sensory data within their context and at generalization with dimensionality reduction (Cauwenberghs, 2013). Perhaps one approach to merging the two systems is to explore where the brain attempts tasks more akin to digital computation, for example in numerical representation (Damarla and Just, 2013).

**Figure 5:**
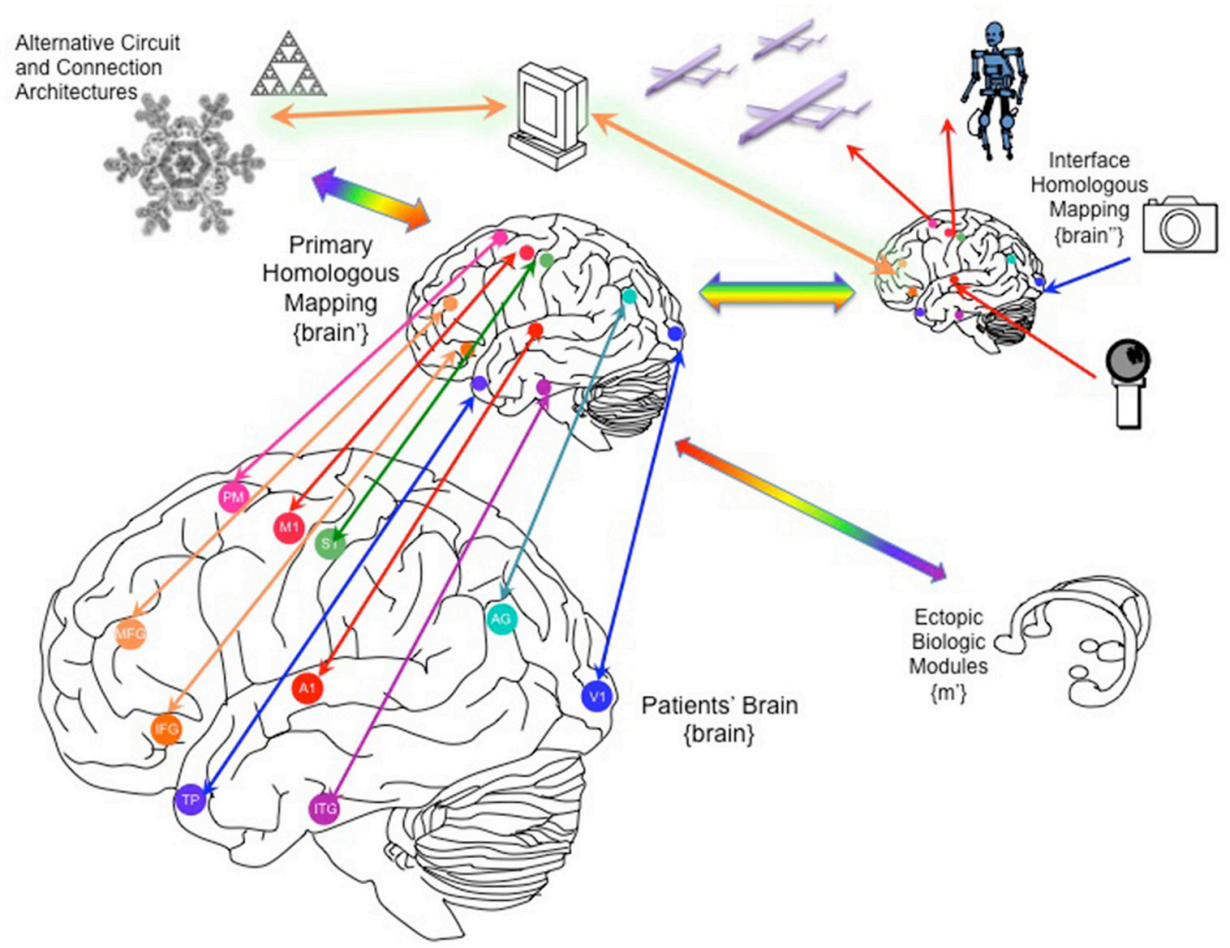
Linking the brain to one or more whole-brain emulations. Through recording/stimulation devices implanted into key primary, heteromodal and higher order cortical targets, the patient's brain can be linked to homologous counterparts in a parallel whole brain emulation that can function as an auxiliary, parallel system. This “primary homologous emulation” can also function as an intermediary to secondary emulations, such as an “interface homologous mapping” explicitly designed to link to its own artificial sensors and effectors, and to ectopic biological modules, including organoids that could be implanted into the patient's peritoneum. In addition to brain-inspired architectures, the primary homologous mapping could be linked to novel artificial architectures derived from other natural organization principles. MFG, middle frotal gyrus; IFG, inferior frontal gyrus; TP, temporal pole, A1, primary auditory cortex, V1, primary visual cortex, S1, primary sensory cortex, M1, primary motor cortex, AG, angular gyrus, PM, premotor cortex.

**Figure 6:**
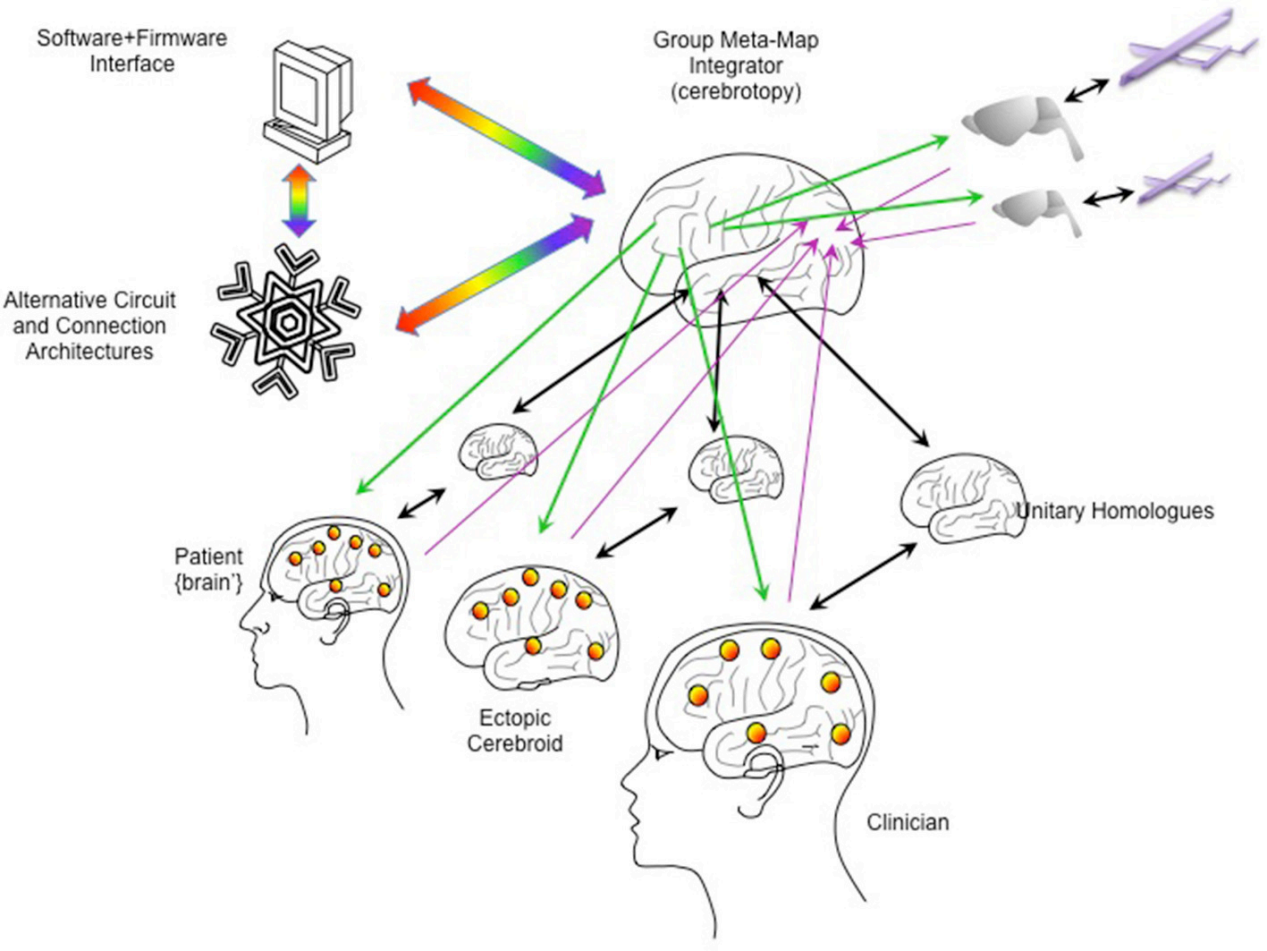
Integrating multiple emulations. Multiple whole brain emulations can be linked together. In this scheme, the patient is linked reciprocally to a homologous emulation (as in Figure 5). An ectopic cerebroid (an organoid construct), itself instrumented with sensor/actuator interfaces, could be implanted into the patient, and could have its own dedicated homologous emulation. A clinician or therapist could guide calibration and rehabilitation through their own BCI interfaces and dedicated homologous. External robotics, such as semi-autonomous drones, could have simpler emulations (here shown as a model on a rodent brain). To coordinate all these emulations, a higher-order “meta-emulation” or “group meta-map integrator” could be forged with input/output connectivity based on principles of how single brains integrate information. Lavender arrows indicate inputs streaming into the “sensory” areas of the integrator, while green arrows indicate outputs streaming from the “motor” areas. Unitary homologs are reciprocally linked to “ventral what stream” temporal cortices, indicated by black arrows. Rainbow arrows indicate connectivity to software/firmware to mediate computer resource use and to explore novel computational architectures.

In addition to the architecture of the “primary emulated brain,” the architecture of secondary, tertiary and numerous other emulations must be considered, and the interconnectivity of these emulations to each other and the real brain (Figure 5). For example, a semi-autonomous drone could be reciprocally linked to an emulation of a fruit fly or other nervous system, and that emulation in turn linked to the human brain emulation (McMains and Somers, 2004; Silver et al., 2007; Haberkern and Jayaraman, 2016). The “mini-emulation” of each drone could drive orienting reflexes within the {midbrain colliculi'} and could interface with real and emulated sensory, prefrontal and cingulo-opercular cortices (Coste and Kleinschmidt, 2016; Zhaoping, 2016). The deployment of multiple emulations may require new “supra-cerebral” constructs to coordinate activity among the emulations and with the “primary real brain.” These “trans-frontal” modeled or bioconstruct cortices would take on a role analogous of the prefrontal cortex within a single human brain: rather than integrating information streams from multiple cortical areas, this “trans-frontal circuit” would need to integrate streams coming from multiple emulations (Figure 6). A WBE offers the possibility of expanding the real estate footprint of cortex beyond the constraints imposed by embryological development and anatomy. If the canonical computational motifs seen throughout mammalian neocortex can be distilled (Turkheimer et al., 2015), could they be replicated in an expanded “sheet” of emulated neocortex itself mapping multiple emulations, and what concrete functions would this provide?

## Summary

The development of novel multi-electrode, optical fiber and biological construct sensor-actuator technologies could allow for the ability to record and stimulate tens of thousands of individual neurons in the human brain across a lifetime. This would in turn present an immediate challenge of how to leverage this massive bandwidth recording/stimulation ability in a clinically meaningful way to benefit children and adults with neurological disease. While simple reconnection strategies that use external electronics to reconnect brain regions may be a starting point (Figure 2), ultimately it may be possible to connect the human brain to itself via a sophisticated, whole-brain emulation, or to multiple such emulations with each one tailored to restoring distinct functions (Figure 5). Along this development pathway, pilot human clinical trials offer an opportunity to begin exploring the parameters of external emulations. Awake, engaged participants can rapidly master new tasks and report subjective experiences, and investigators can likewise leverage machine-learning algorithms and advanced neuroimaging to accelerate the emulation parameter optimization. The whole brain emulation could provide patients an auxiliary, parallel mirror brain system that could intrinsically compensate for dysfunction within their own original brain. Likewise, the whole brain emulation could give rise to intelligent abilities in itself and this phenomenon could depend on its entrainment to a real human being. “Whole brain emulations” could hence move from being large-scale brain models designed as a facsimile of reality, into advanced medical devices and engineering tools that are part of a new reality to benefit human health and wellbeing.

## Author contributions

MS is responsible for all content and writing of this manuscript, including creation of figures.

### Conflict of interest statement

The author declares that the research was conducted in the absence of any commercial or financial relationships that could be construed as a potential conflict of interest.

## Footnotes

^1^brain-map.org.2017,~“brain-map.org.” Available online at: www.brain-map.org SFN Brain Map, 2017 “SFN Brain Map.” INCF, 2017 “INCF.” Available online at: www.incf.org
